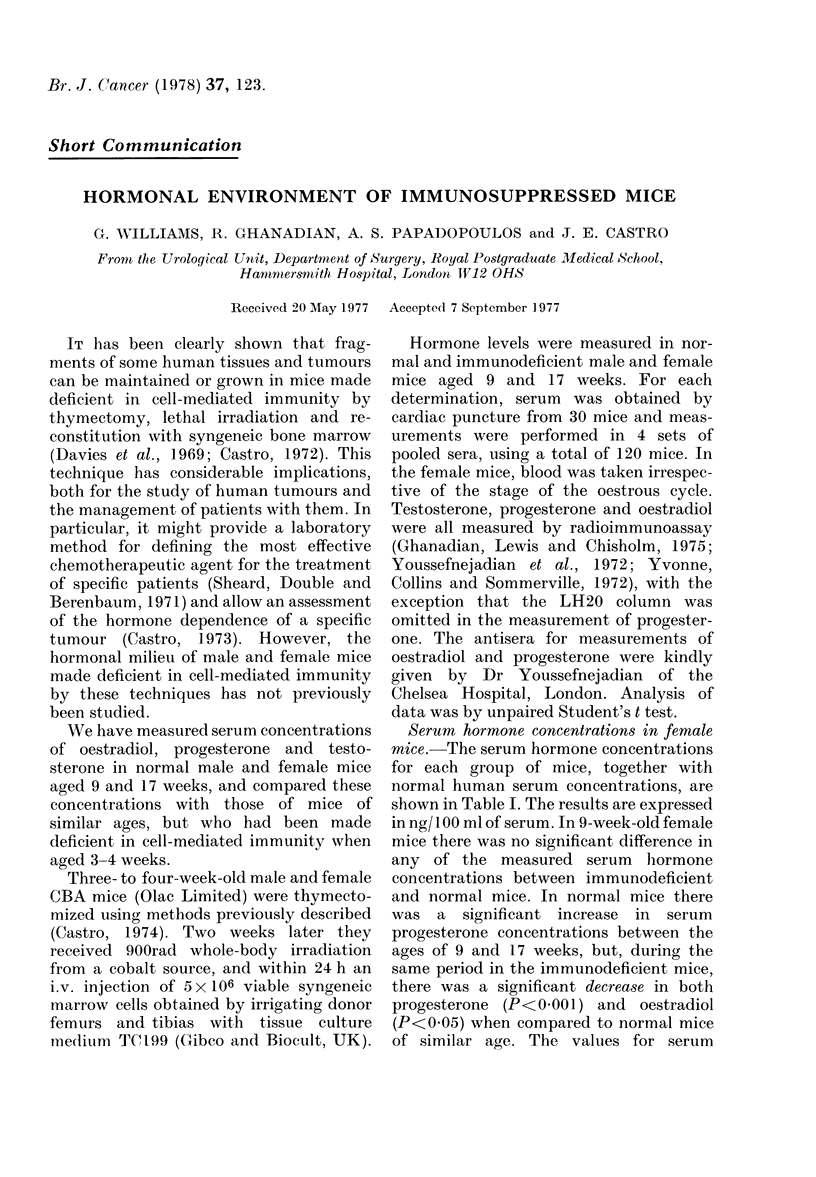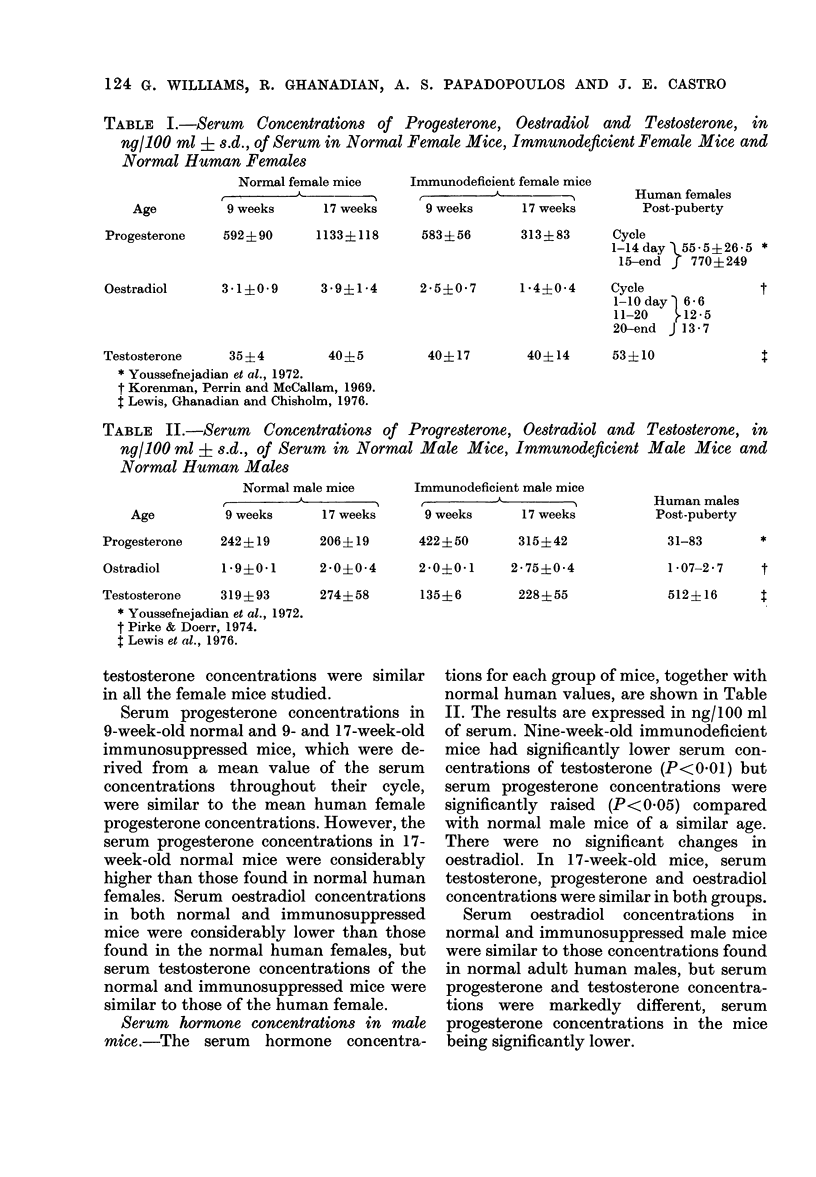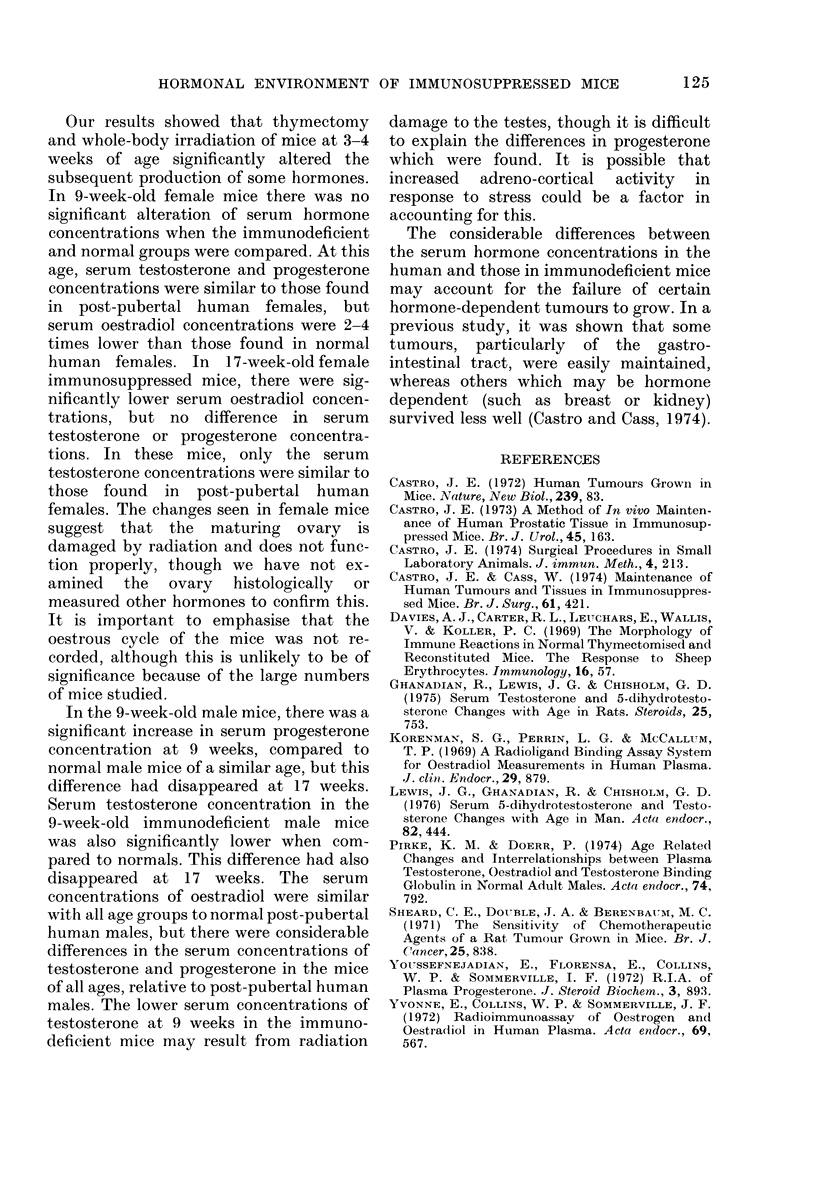# Hormonal environment of immunosuppressed mice.

**DOI:** 10.1038/bjc.1978.17

**Published:** 1978-01

**Authors:** G. Williams, R. Ghanadian, A. S. Papadopoulos, J. E. Castro


*Br. J. Cancer* (1978) **37,** 123.

***Short Communication***

### HORMONAL ENVIRONMENT OF IMMUNOSUPPRESSED MICE

G. WILLIAMS, R. GHANADIAN, A. S. PAPADOPOULOS and J. E. CASTRO

*From the Urological Unit, Department of Surgery, Royal Postgraduate Medical School, Hammersmith Hospital, London W12 OHS*

Received 20 May 1977 Accepted 7 September 1977

IT has been clearly shown that fragments of some human tissues and tumours can be maintained or grown in mice made deficient in cell-mediated immunity by thymectomy, lethal irradiation and reconstitution with syngeneic bone marrow (Davies *et al.*, 1969; Castro, 1972). This technique has considerable implications, both for the study of human tumours and the management of patients with them. In particular, it might provide a laboratory method for defining the most effective chemotherapeutic agent for the treatment of specific patients (Sheard, Double and Berenbaum, 1971) and allow an assessment of the hormone dependence of a specific tumour (Castro, 1973). However, the hormonal milieu of male and female mice made deficient in cell-mediated immunity by these techniques has not previously been studied.

We have measured serum concentrations of oestradiol, progesterone and testosterone in normal male and female mice aged 9 and 17 weeks, and compared these concentrations with those of mice of similar ages, but who had been made deficient in cell-mediated immunity when aged 3–4 weeks.

Three- to four-week-old male and female CBA mice (Olac Limited) were thymectomized using methods previously described (Castro, 1974). Two weeks later they received 900rad whole-body irradiation from a cobalt source, and within 24 h an i.v. injection of $5\times10^6$ viable syngeneic marrow cells obtained by irrigating donor femurs and tibias with tissue culture medium TC199 (Gibco and Biocult, UK).

Hormone levels were measured in normal and immunodeficient male and female mice aged 9 and 17 weeks. For each determination, serum was obtained by cardiac puncture from 30 mice and measurements were performed in 4 sets of pooled sera, using a total of 120 mice. In the female mice, blood was taken irrespective of the stage of the oestrous cycle. Testosterone, progesterone and oestradiol were all measured by radioimmunoassay (Ghanadian, Lewis and Chisholm, 1975; Youssefnejadian *et al.*, 1972; Yvonne, Collins and Sommerville, 1972), with the exception that the LH20 column was omitted in the measurement of progesterone. The antisera for measurements of oestradiol and progesterone were kindly given by Dr Youssefnejadian of the Chelsea Hospital, London. Analysis of data was by unpaired Student's *t* test.

*Serum hormone concentrations in female mice.*—The serum hormone concentrations for each group of mice, together with normal human serum concentrations, are shown in Table I. The results are expressed in ng/100 ml of serum. In 9-week-old female mice there was no significant difference in any of the measured serum hormone concentrations between immunodeficient and normal mice. In normal mice there was a significant increase in serum progesterone concentrations between the ages of 9 and 17 weeks, but, during the same period in the immunodeficient mice, there was a significant *decrease* in both progesterone ($P<0{\cdot}001$) and oestradiol ($P<0{\cdot}05$) when compared to normal mice of similar age. The values for serum

TABLE I.—*Serum Concentrations of Progesterone, Oestradiol and Testosterone, in ng/100 ml ± s.d., of Serum in Normal Female Mice, Immunodeficient Female Mice and Normal Human Females*

| | Normal female mice | | Immunodeficient female mice | | Human females | |
|---|---|---|---|---|---|---|
| Age | 9 weeks | 17 weeks | 9 weeks | 17 weeks | Post-puberty | |
| Progesterone | 592±90 | 1133±118 | 583±56 | 313±83 | Cycle<br>1–14 day 55·5±26·5<br>15–end 770±249 | * |
| Oestradiol | 3·1±0·9 | 3·9±1·4 | 2·5±0·7 | 1·4±0·4 | Cycle<br>1–10 day 6·6<br>11–20 12·5<br>20–end 13·7 | † |
| Testosterone | 35±4 | 40±5 | 40±17 | 40±14 | 53±10 | ‡ |

* Youssefnejadian *et al.*, 1972.
† Korenman, Perrin and McCallam, 1969.
‡ Lewis, Ghanadian and Chisholm, 1976.

TABLE II.—*Serum Concentrations of Progresterone, Oestradiol and Testosterone, in ng/100 ml ± s.d., of Serum in Normal Male Mice, Immunodeficient Male Mice and Normal Human Males*

| | Normal male mice | | Immunodeficient male mice | | Human males | |
|---|---|---|---|---|---|---|
| Age | 9 weeks | 17 weeks | 9 weeks | 17 weeks | Post-puberty | |
| Progesterone | 242±19 | 206±19 | 422±50 | 315±42 | 31–83 | * |
| Ostradiol | 1·9±0·1 | 2·0±0·4 | 2·0±0·1 | 2·75±0·4 | 1·07–2·7 | † |
| Testosterone | 319±93 | 274±58 | 135±6 | 228±55 | 512±16 | ‡ |

* Youssefnejadian *et al.*, 1972.

† Pirke & Doerr, 1974.

‡ Lewis *et al.*, 1976.

testosterone concentrations were similar in all the female mice studied.

Serum progesterone concentrations in 9-week-old normal and 9- and 17-week-old immunosuppressed mice, which were derived from a mean value of the serum concentrations throughout their cycle, were similar to the mean human female progesterone concentrations. However, the serum progesterone concentrations in 17-week-old normal mice were considerably higher than those found in normal human females. Serum oestradiol concentrations in both normal and immunosuppressed mice were considerably lower than those found in the normal human females, but serum testosterone concentrations of the normal and immunosuppressed mice were similar to those of the human female.

*Serum hormone concentrations in male mice.*—The serum hormone concentrations for each group of mice, together with normal human values, are shown in Table II. The results are expressed in ng/100 ml of serum. Nine-week-old immunodeficient mice had significantly lower serum concentrations of testosterone ($P<0{\cdot}01$) but serum progesterone concentrations were significantly raised ($P<0{\cdot}05$) compared with normal male mice of a similar age. There were no significant changes in oestradiol. In 17-week-old mice, serum testosterone, progesterone and oestradiol concentrations were similar in both groups.

Serum oestradiol concentrations in normal and immunosuppressed male mice were similar to those concentrations found in normal adult human males, but serum progesterone and testosterone concentrations were markedly different, serum progesterone concentrations in the mice being significantly lower.

Our results showed that thymectomy and whole-body irradiation of mice at 3–4 weeks of age significantly altered the subsequent production of some hormones. In 9-week-old female mice there was no significant alteration of serum hormone concentrations when the immunodeficient and normal groups were compared. At this age, serum testosterone and progesterone concentrations were similar to those found in post-pubertal human females, but serum oestradiol concentrations were 2–4 times lower than those found in normal human females. In 17-week-old female immunosuppressed mice, there were significantly lower serum oestradiol concentrations, but no difference in serum testosterone or progesterone concentrations. In these mice, only the serum testosterone concentrations were similar to those found in post-pubertal human females. The changes seen in female mice suggest that the maturing ovary is damaged by radiation and does not function properly, though we have not examined the ovary histologically or measured other hormones to confirm this. It is important to emphasise that the oestrous cycle of the mice was not recorded, although this is unlikely to be of significance because of the large numbers of mice studied.

In the 9-week-old male mice, there was a significant increase in serum progesterone concentration at 9 weeks, compared to normal male mice of a similar age, but this difference had disappeared at 17 weeks. Serum testosterone concentration in the 9-week-old immunodeficient male mice was also significantly lower when compared to normals. This difference had also disappeared at 17 weeks. The serum concentrations of oestradiol were similar with all age groups to normal post-pubertal human males, but there were considerable differences in the serum concentrations of testosterone and progesterone in the mice of all ages, relative to post-pubertal human males. The lower serum concentrations of testosterone at 9 weeks in the immunodeficient mice may result from radiation damage to the testes, though it is difficult to explain the differences in progesterone which were found. It is possible that increased adreno-cortical activity in response to stress could be a factor in accounting for this.

The considerable differences between the serum hormone concentrations in the human and those in immunodeficient mice may account for the failure of certain hormone-dependent tumours to grow. In a previous study, it was shown that some tumours, particularly of the gastro-intestinal tract, were easily maintained, whereas others which may be hormone dependent (such as breast or kidney) survived less well (Castro and Cass, 1974).

### REFERENCES

Castro, J. E. (1972) Human Tumours Grown in Mice. *Nature, New Biol.*, **239**, 83.

Castro, J. E. (1973) A Method of *In vivo* Maintenance of Human Prostatic Tissue in Immunosuppressed Mice. *Br. J. Urol.*, **45**, 163.

Castro, J. E. (1974) Surgical Procedures in Small Laboratory Animals. *J. immun. Meth.*, **4**, 213.

Castro, J. E. & Cass, W. (1974) Maintenance of Human Tumours and Tissues in Immunosuppressed Mice. *Br. J. Surg.*, **61**, 421.

Davies, A. J., Carter, R. L., Leuchars, E., Wallis, V. & Koller, P. C. (1969) The Morphology of Immune Reactions in Normal Thymectomised and Reconstituted Mice. The Response to Sheep Erythrocytes. *Immunology*, **16**, 57.

Ghanadian, R., Lewis, J. G. & Chisholm, G. D. (1975) Serum Testosterone and 5-dihydrotestosterone Changes with Age in Rats. *Steroids*, **25**, 753.

Korenman, S. G., Perrin, L. G. & McCallum, T. P. (1969) A Radioligand Binding Assay System for Oestradiol Measurements in Human Plasma. *J. clin. Endocr.*, **29**, 879.

Lewis, J. G., Ghanadian, R. & Chisholm, G. D. (1976) Serum 5-dihydrotestosterone and Testosterone Changes with Age in Man. *Acta endocr.*, **82**, 444.

Pirke, K. M. & Doerr, P. (1974) Age Related Changes and Interrelationships between Plasma Testosterone, Oestradiol and Testosterone Binding Globulin in Normal Adult Males. *Acta endocr.*, **74**, 792.

Sheard, C. E., Double, J. A. & Berenbaum, M. C. (1971) The Sensitivity of Chemotherapeutic Agents of a Rat Tumour Grown in Mice. *Br. J. Cancer*, **25**, 838.

Youssefnejadian, E., Florensa, E., Collins, W. P. & Sommerville, I. F. (1972) R.I.A. of Plasma Progesterone. *J. Steroid Biochem.*, **3**, 893.

Yvonne, E., Collins, W. P. & Sommerville, J. F. (1972) Radioimmunoassay of Oestrogen and Oestradiol in Human Plasma. *Acta endocr.*, **69**, 567.